# Perioperative immunochemotherapy for resectable gastric or gastroesophageal junction cancer: a meta-analysis of efficacy and safety from randomized trials

**DOI:** 10.3389/fimmu.2026.1765780

**Published:** 2026-08-25

**Authors:** Juan Xu, Xiao-mei Li, Chen Yang, Rui Zhang, Hai-lin Lyu, Li Wang, Jing Xie

**Affiliations:** 1 Beijing Shi Jingshan Cadre Sanatorium of the Beijing Garrison, Beijing, China; 2 Laoshan Medical District of No. 971 Hospital of Chinese Navy, Qingdao, Shandong, China; 3 Department of Emergency Medicine, Rocket Force Characteristic Medical Center, Beijing, China; 4 Department of Oncology, Sunshine Union Hospital, Weifang, Shandong, China; 5 Department of Gastroenterology, Rocket Force Characteristic Medical Center, Beijing, China

**Keywords:** chemotherapy, gastric or gastroesophageal cancer, immune checkpoint inhibitors, meta-analysis, perioperative, randomized controlled trials

## Abstract

**Background:**

The efficacy and safety of perioperative immune checkpoint inhibitors (ICIs) combined with chemotherapy for resectable gastric/gastroesophageal junction (GEJ) cancer remain to be definitively established. This meta-analysis aimed to synthesize the latest evidence from randomized controlled trials (RCTs) on this emerging therapeutic strategy.

**Methods:**

A systematic literature search was conducted in PubMed, Embase, and the Cochrane Library up to June 2026 for RCTs comparing perioperative ICI plus chemotherapy with chemotherapy alone in patients with resectable gastric/GEJ cancer. Primary outcomes included pathological complete response (pCR), R0 resection rate, and survival endpoints (event-free survival [EFS] and overall survival [OS]). Safety was assessed by the incidence of adverse events. Pooled risk ratios (RRs), hazard ratios (HRs), and 95% confidence intervals (CIs) were calculated using random-effects models.

**Results:**

Seven RCTs comprising 3252 patients were included. The addition of ICIs to chemotherapy significantly improved the pCR rate compared to chemotherapy alone (RR: 2.91, 95% CI: 2.16 to 3.93, *P* < 0.001). The ICI-chemotherapy combination also significantly prolonged EFS (HR: 0.74, 95% CI: 0.66 to 0.83, *P* < 0.001) and OS (HR: 0.80, 95% CI: 0.66 to 0.95, *P* = 0.013). Exploratory subgroup analyses suggested numerically greater pCR benefit in patients with PD-L1 positive expression (CPS ≥1) or microsatellite instability high (MSI-H) tumors. The R0 resection rate was similar between the two groups. Moreover, the incidences of any-grade and severe adverse events showed no statistically significant difference between the two groups.

**Conclusion:**

This meta-analysis provides updated evidence that the addition of ICIs to perioperative chemotherapy significantly enhances pathological responses and survival outcomes in patients with resectable gastric/GEJ cancer, with a safety profile that showed no statistically significant difference in adverse events compared to chemotherapy alone. These results further reinforce the use of perioperative ICI-chemotherapy combination as a promising treatment strategy for this patient population, although confirmation in larger trials with longer follow-up is warranted.

## 1 Introduction

Gastric and gastroesophageal junction (GEJ) cancers constitute a major global health burden and remain among the leading causes of cancer-related mortality worldwide (1). For patients with locally advanced, resectable disease, surgical resection represents the primary curative modality. However, a significant proportion of patients experience disease recurrence following surgery (2). The adoption of perioperative chemotherapy (neoadjuvant and/or adjuvant chemotherapy) has been established as a standard strategy to mitigate recurrence risk and improve survival outcomes (3–6). Despite this advancement, long-term results remain unsatisfactory, with many patients still succumbing to the disease, thereby underscoring the urgent need for more effective therapeutic strategies.

Recent years have witnessed a paradigm shift in the management of advanced gastric/GEJ cancers with the introduction of immune checkpoint inhibitors (ICIs). The combination of ICIs with conventional chemotherapy has demonstrated significant improvements in survival outcomes in the metastatic setting (7, 8), offering a new therapeutic backbone. Building upon this success, several studies have been initiated to evaluate whether integrating ICIs into perioperative treatment regimens can yield similar benefits for patients with resectable disease (9, 10). However, the reported results from these trials have been heterogeneous, with some demonstrating marked improvements while others showing more modest benefits, creating clinical uncertainty.

To address this uncertainty, we performed a meta-analysis of all relevant randomized controlled trials (RCTs) comparing perioperative ICI-chemotherapy combination therapy with chemotherapy alone in patients with resectable gastric or GEJ cancer. Our objective was to precisely evaluate the efficacy and safety of this combination, thereby offering a comprehensive assessment of its clinical value.

## 2 Materials and methods

### 2.1 Search strategy

A systematic literature search was conducted up to November 17, 2025 across PubMed, Embase, and the Cochrane Central Register of Controlled Trials (CENTRAL). During the revision process, we updated this search to June 30, 2026 to incorporate the most recent evidence, including updated survival data from major conferences and newly published long-term follow-up results. The strategy incorporated key terms related to gastric cancer, gastroesophageal junction cancer, perioperative therapy, and immune checkpoint inhibitors (e.g., PD-1, PD-L1, CTLA-4). The search was restricted to publications in English. In addition, we manually screened the proceedings of major international conferences to identify updated survival data not yet available in full-text publications. Relevant references from the included studies and recent review articles were also hand-searched to identify additional eligible trials. The specific search syntax for each database is detailed in **Supplementary Tables 1–6**. Our objective was to identify RCTs that evaluated the addition of ICIs to chemotherapy versus chemotherapy alone as perioperative treatment for resectable gastric or GEJ cancer. The methodology for this meta-analysis adhered to the Preferred Reporting Items for Systematic Reviews and Meta-Analyses (PRISMA) guidelines (11).

### 2.2 Selection criteria

The study selection was independently performed by two investigators. Included RCTs were required to compare perioperative chemotherapy combined with ICIs against chemotherapy alone in patients with resectable gastric or GEJ cancer. Additionally, trials must have reported on at least one of the predefined efficacy endpoints, which comprised pathological complete response (pCR), R0 resection rate, event-free survival (EFS), and overall survival (OS).

pCR was defined as the absence of viable tumor cells in both the primary tumor and sampled lymph nodes. EFS was measured from randomization to the first occurrence of disease progression, recurrence, or death from any cause. OS was defined as the time from randomization to death from any cause. The R0 resection rate referred to the proportion of patients achieving a microscopically margin-negative resection.

Safety was assessed by the incidence of any-grade and severe (grade ≥3) adverse events (AEs), graded according to the Common Terminology Criteria for Adverse Events. Any discrepancies between reviewers were resolved through consensus.

### 2.3 Data extraction

Two investigators independently performed data extraction. The extracted dataset encompassed the following key aspects: reference details (including trial name, publication year, study design, and phase), patient characteristics (specifically diagnosis, tumor location, PD-L1 expression status, microsatellite instability status, and sample size), and detailed treatment regimens (documenting both the experimental ICI plus chemotherapy protocols, specifying the ICI drug, administration frequencies, and treatment cycles, and the control chemotherapy regimens). All predefined efficacy and safety outcomes were systematically collected. For time-to-event outcomes (EFS and OS), hazard ratios (HRs) and their corresponding 95% confidence intervals (CIs) were extracted directly from the original publications or **Supplementary Materials**; no digitization software was used to approximate values from Kaplan-Meier curves. Any discrepancies identified during this process were resolved through consensus discussion, with correspondence initiated to the authors of the primary studies for clarification when necessary.

### 2.4 Methodological quality evaluation

The methodological quality of the included RCTs was rigorously evaluated using the Cochrane Risk of Bias tool (12). Two reviewers independently assessed seven critical domains: adequacy of the randomization process, allocation concealment, blinding of participants and personnel, blinding of outcome assessment, handling of incomplete outcome data, potential for selective reporting, and other possible sources of bias. For each domain, a judgment of “low risk,” “high risk,” or “unclear risk” was assigned based on the criteria outlined in the Cochrane Handbook for Systematic Reviews of Interventions.

### 2.5 Statistical analyses

All statistical analyses were performed using Stata 12.0 (Stata Corporation, College Station, TX, USA). Time-to-event outcomes were summarized as HRs with 95% CIs, while dichotomous outcomes were expressed as risk ratios (RRs) with 95% CIs. For all meta-analyses, we employed the inverse variance method under a random-effects model (DerSimonian-Laird estimator). This approach assigns weights to each study based on the inverse of its variance, providing conservative effect estimates irrespective of the observed heterogeneity. Heterogeneity among studies was assessed using the *I^2^* statistic, with *I^2^* > 50% and a *P*-value < 0.10 indicating substantial heterogeneity (13). To address potential heterogeneity sources, we performed subgroup and sensitivity analyses where feasible. A two-sided *P*-value < 0.05 was considered statistically significant for all tests.

## 3 Results

### 3.1 Study selection and characteristics

In the initial search (up to November 17, 2025), the systematic literature search yielded 1920 potentially relevant records. After removing duplicates, 1653 records were screened by title and abstract, of which 1634 were excluded. The remaining 19 full-text articles were assessed for eligibility, resulting in the exclusion of 14 studies for reasons detailed in **Supplementary Table 7**. Through this initial search, five RCTs (14–18) were included in the meta-analysis.

In the updated search (November 18, 2025 to June 30, 2026), the systematic search identified 472 potentially relevant records. After removing duplicates, 418 records were screened by title and abstract, leading to the exclusion of 404 records. The remaining 14 full-text articles were assessed for eligibility, of which 10 were excluded (see **Supplementary Table 8** for reasons). Of the four remaining records, two were updated survival analyses of trials already included in the initial search (19, 20), and two were newly identified RCTs (21, 22).

**Figure 1:**
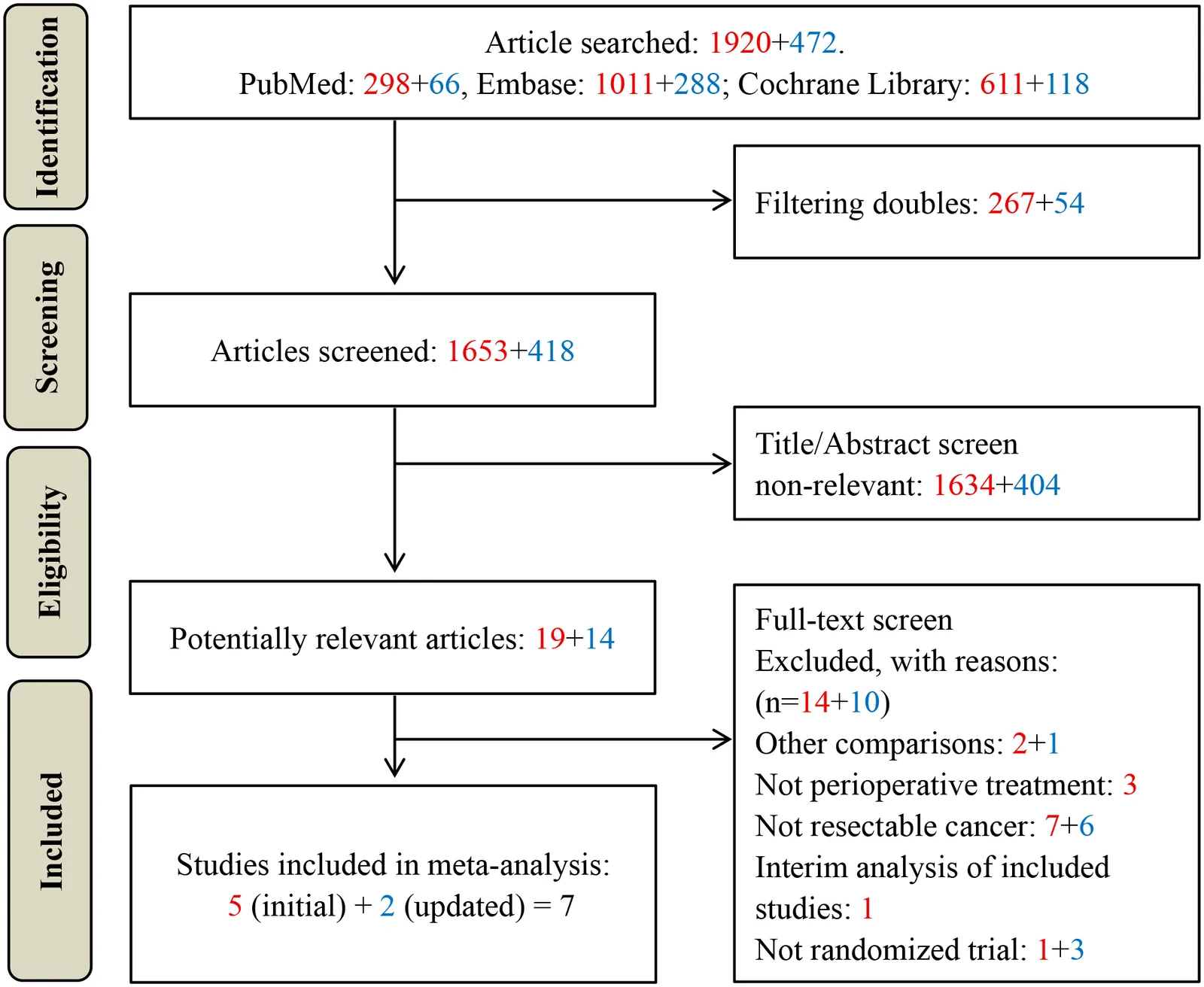
Study selection flow diagram. Results from the initial search (up to November 17, 2025) are shown in red, and results from the updated search (November 18, 2025 to June 30, 2026) are shown in blue.

In total, seven RCTs were included in the final meta-analysis, involving 3252 patients (1620 in the ICI plus chemotherapy group and 1632 in the chemotherapy-alone group). This selection process is summarized in **Figure 1**.

The characteristics of the seven included RCTs are summarized in **Tables 1** and **2**. Sample sizes ranged from 42 to 1007 patients. The cohort consisted of three Phase III and four Phase II trials, with all seven being multicenter studies. In terms of intervention, four trials utilized a PD-1 inhibitor (pembrolizumab, toripalimab, serplulimab, or sintilimab), while three employed a PD-L1 inhibitor (atezolizumab in two trials and durvalumab in one). The chemotherapy backbones varied: two trials used the FLOT regimen, two used SOX, one allowed a choice of FP/XP/FLOT, one used XELOX, and the remaining trial used SOX or XELOX.

**Table 1:** Summary characteristics of the included randomized controlled trials.

Study references	Publication year	Study design	Phase	Sample sizes	Diagnosis	ICI drug (target)	Intervention group	Control group	Study endpoints
Shitara et al. (15)	2025	Multicenter RCT	III	502/505	Resectable locally advanced gastric or GEJ cancer	Pembrolizumab (PD-1)	Pembrolizumab plus FP/XP/FLOT for 6 cycles (3 preoperative and 3 postoperative), followed by 11 cycles of pembrolizumab monotherapy	Placebo plus FP/XP/FLOT for 6 cycles (3 preoperative and 3 postoperative), followed by 11 cycles of placebo monotherapy	pCR, EFS, OS
Janjigian et al. (18, 19)	2025	Multicenter RCT	III	474/474	Resectable gastric or GEJ cancer	Durvalumab (PD-L1)	Durvalumab plus FLOT for 4 cycles (2 preoperative and 2 postoperative), followed by durvalumab for 10 cycles	Placebo plus FLOT for 4 cycles (2 preoperative and 2 postoperative), followed by placebo for 10 cycles	pCR, R0 resection rate, EFS, OS
Peng et al. (16)	2025	Multicenter RCT	II	21/21	Resectable locally advanced gastric or GEJ cancer	Atezolizumab (PD-L1)	Atezolizumab plus XELOX plus trastuzumab for 8 cycles (3 preoperative and 5 postoperative)	XELOX plus trastuzumab for 8 cycles (3 preoperative and 5 postoperative)	pCR, R0 resection rate
Lorenzen et al. (17)	2024	Multicenter RCT	II	146/149	Resectable gastric or GEJ cancer	Atezolizumab (PD-L1)	Atezolizumab plus FLOT for 8 cycles (4 preoperative and 4 postoperative), followed by 8 cycles of atezolizumab maintenance	FLOT for 8 cycles (4 preoperative and 4 postoperative)	pCR, R0 resection rate
Yuan et al. (14, 20)	2024	Multicenter RCT	II	54/54	Resectable locally advanced gastric or GEJ cancer	Toripalimab (PD-1)	Toripalimab plus SOX/XELOX for 8 cycles (3 preoperative and 5 postoperative), followed by toripalimab monotherapy for up to 6 months	SOX/XELOX for 8 cycles (3 preoperative and 5 postoperative)	pCR, R0 resection rate
Shen et al. (21)	2026	Multicenter RCT	III	292/296	Resectable gastric or GEJ cancer (PD-L1-positive)	Serplulimab (PD-1)	Serplulimab plus SOX for 3 cycles, followed by adjuvant serplulimab for 17 cycles	Placebo plus SOX for 3 cycles, followed by adjuvant SOX for 5 cycles	pCR, R0 resection rate, EFS
Ding et al. (22)	2025	Multicenter RCT	II	131/133	Resectable locally advanced gastric or GEJ cancer	Sintilimab (PD-1)	Sintilimab plus SOX for 8 cycles (3 preoperative and 5 postoperative), followed by sintilimab monotherapy for up to 1 year	SOX for 8 cycles (3 preoperative and 5 postoperative)	pCR

ICI, immune checkpoint inhibitor; RCT, randomized controlled trial; GEJ, gastroesophageal junction; FLOT, fluorouracil, leucovorin, oxaliplatin, and docetaxel; FP, cisplatin and fluorouracil; XP, cisplatin and capecitabine; SOX, S-1 and oxaliplatin; XELOX, capecitabine and oxaliplatin; pCR, pathological complete response; EFS, event-free survival; OS, overall survival.

**Table 2:** Patient characteristics for the included randomized controlled trials.

Study references	Tumor location		PD-L1 expression		Microsatellite status		Follow-up period
	ICI group	Control group	ICI group	Control group	ICI group	Control group	
Shitara et al. (15)	Gastric: 376 (75%), gastroesophageal junction: 126 (25%)	Gastric: 386 (76%), gastroesophageal junction: 118 (23%), missing: 1 (<1)	CPS<1: 91 (18%), CPS≥1: 366 (73%), not evaluable: 32 (6%), missing: 13 (3%)	CPS<1: 91 (18%), CPS≥1: 384 (76%), not evaluable: 21 (4%), missing: 9 (2%)	MSI high: 43 (9%), not high: 390 (78%), not evaluable: 49 (10%), missing: 20 (4%)	MSI high: 38 (8%), not high: 382 (76%), not evaluable: 70 (14%), missing: 15 (3%)	59.9 (39.0-75.8) months
Janjigian et al. (18, 19)	Gastric: 324 (68.4%), gastroesophageal junction: 150 (31.6%)	Gastric: 316 (66.7%), gastroesophageal junction: 158 (33.3%)	TAP<1%: 48 (10.1%), TAP≥1%: 426 (89.9%)	TAP <1%: 47 (9.9%), TAP≥1%: 427 (90.1%)	MSI high: 25 (5.3%), not high: 301 (63.5%), not evaluable: 69 (14.6%), missing: 79 (16.7%)	MSI high: 24 (5.1%), not high: 310 (65.4%), not evaluable: 52 (11.0%), missing: 88 (18.6%)	31.5 (26.7-36.6) months
Peng et al. (16)	Gastric: 12 (57%), gastroesophageal junction: 5 (24%), whole gastric: 3 (14%), other: 1 (5%)	Gastric: 14 (67%), gastroesophageal junction: 4 (19%), whole gastric: 2 (10%), other: 1 (5%)	CPS≥1: 15 (71%), CPS<1: 3 (14%), unknown: 3 (14%)	CPS≥1: 14 (67%), CPS<1: 4 (19%), unknown: 3 (14%)	NA	NA	15.7 months
Lorenzen et al. (17)	Gastric: 56 (38%), gastroesophageal junction: 90 (62%)	Gastric: 58 (39%), gastroesophageal junction: 91 (61%)	CPS<1: 64 (44%), CPS≥1: 82 (56%), CPS≥5: 40 (27%), CPS≥10: 27 (19%)	CPS<1: 58 (39%), CPS≥1: 88 (59%), CPS≥5: 41 (28%), CPS≥10: 26 (17%), missing: 3 (2%)	MSI/dMMR: 8 (6%), MSS/pMMR: 138 (95%)	MSI/dMMR: 15 (10%), MSS/pMMR: 134 (90%)	NA
Yuan et al. (14, 20)	Gastric: 37 (68.5%), gastroesophageal junction: 17 (31.5%)	Gastric: 34 (63.0%), gastroesophageal junction: 20 (37.0%)	CPS<5: 32 (59.3%), CPS≥5: 11 (20.4%), not evaluable: 11 (20.4%)	CPS<5: 39 (72.2%), CPS≥5: 6 (11.1%), not evaluable: 9 (16.7%)	pMMR: 47 (87.0%), dMMR: 3 (5.6%), NA: 4 (7.4%)	pMMR: 46 (85.2%), dMMR: 3 (5.6%), NA: 5 (9.3%)	43.2 months
Shen et al. (21)	NA	NA	CPS≥5: 292 (100%)	CPS≥5: 296 (100%)	NA	NA	35.9 (23.5-49.4) months
Ding et al. (22)	NA	NA	NA	NA	NA	NA	NA

ICI, immune checkpoint inhibitor; TAP, tumor area positivity; CPS, combined positive score; MSI, microsatellite instability; MSS, microsatellite stability; d/pMMR, deficient/proficient mismatch repair; NA, not available.

### 3.2 Methodological quality evaluation

Six of the seven included RCTs adequately described their randomization procedures (14–18, 21); the remaining trial was reported as a conference abstract (22) and did not provide sufficient detail on randomization. Allocation concealment was implemented in five RCTs (15–18), but was not reported in the remaining two (14, 22). The blinding methods varied: three trials were double-blind (15, 18, 21), two reported that pathologic assessment was performed by evaluators blinded to the treatment assignment (14, 17), and two were conducted as open-label studies (16, 22). For the remaining quality domains: incomplete outcome data, selective reporting, and other potential biases, six studies were assessed as having a low risk of bias (14–18, 21); the remaining trial (22) was not assessed due to insufficient information in the conference abstract. The comprehensive risk-of-bias assessment is detailed in **Supplementary Figure 1**.

### 3.3 Pathological complete response

**Figure 2:**
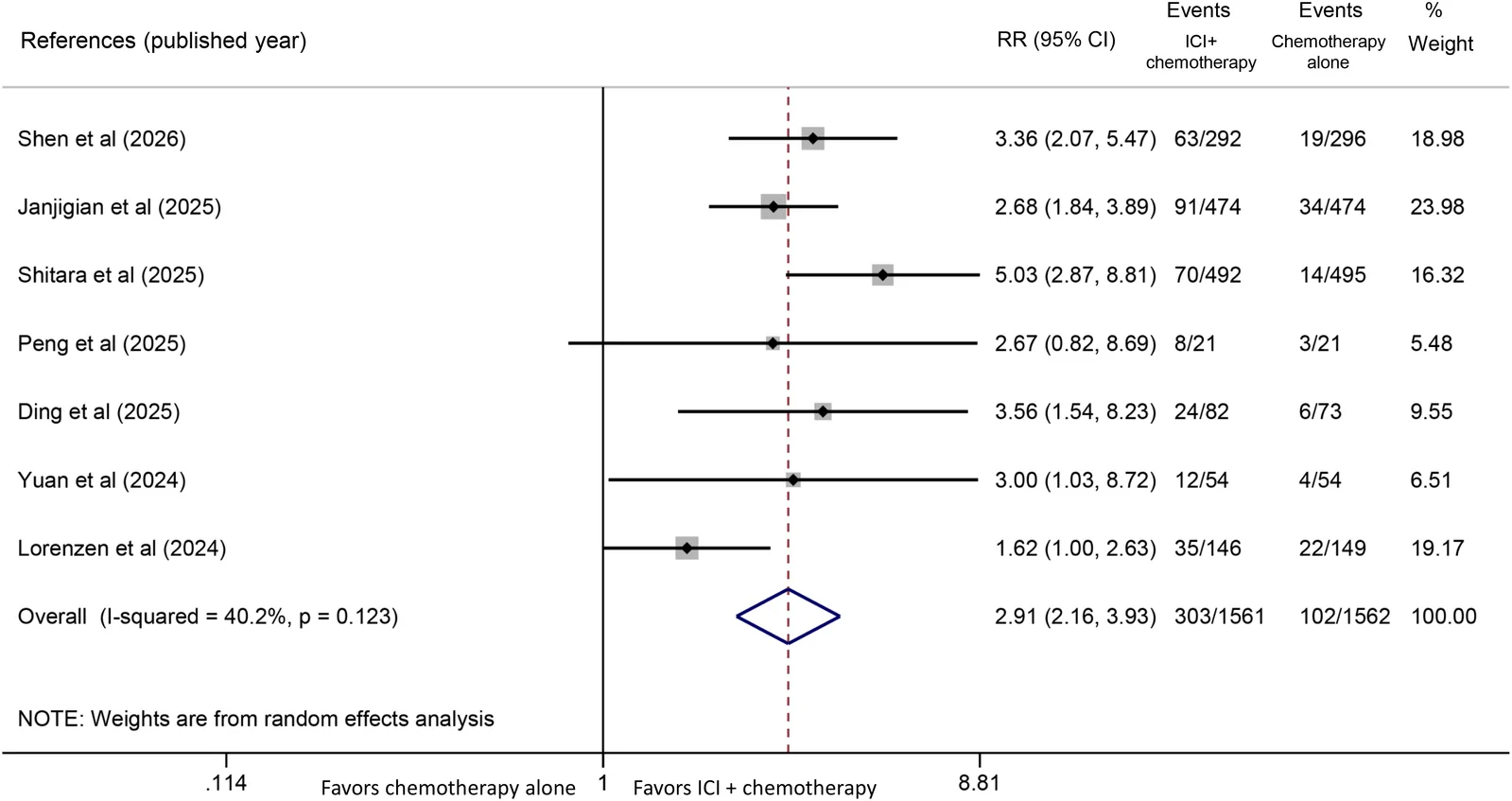
Forest plot of pathological response rates comparing perioperative ICIs plus chemotherapy versus chemotherapy alone in patients with resectable gastric/gastroesophageal junction cancer. ICIs, immune checkpoint inhibitors. RR >1 favors ICIs plus chemotherapy.

Pathological complete response was reported in all seven included studies (14–18, 21, 22). Pooled analysis demonstrated that the combination of ICI and chemotherapy provided a directionally consistent improvement in pCR rates compared to chemotherapy alone (RR: 2.91, 95% CI: 2.16 to 3.93, *P* < 0.001), with moderate heterogeneity observed (*I²* = 40.2%) (**Figure 2**).

**Figure 3:**
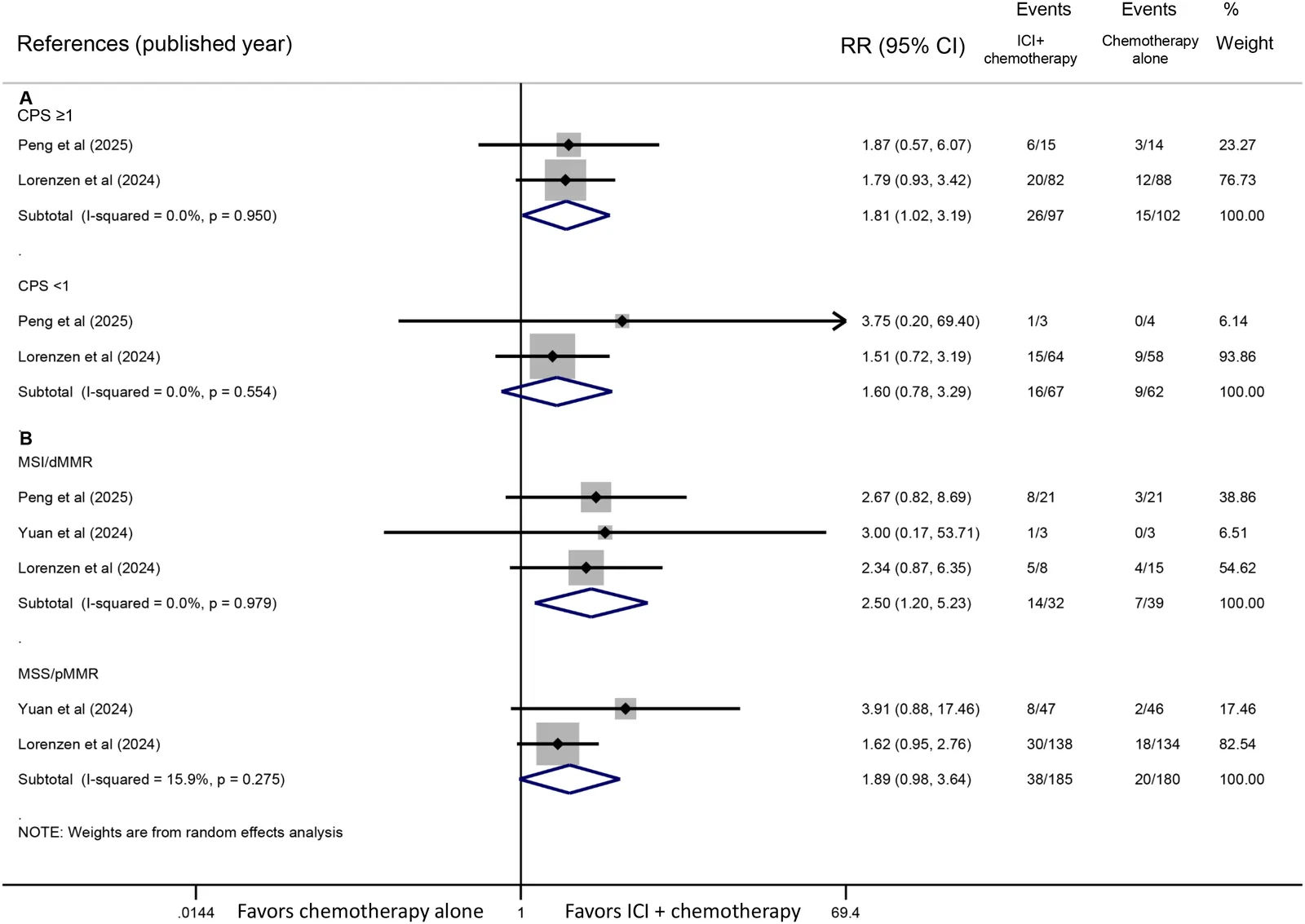
Subgroup analyses of pathological response benefits from perioperative ICIs plus chemotherapy stratified by: **(A)** PD-L1 expression and **(B)** microsatellite status. ICIs, immune checkpoint inhibitors. CPS, combined positive score; MSI, microsatellite instability; MSS, microsatellite stability; d/pMMR, deficient/proficient mismatch repair. RR >1 favors ICIs plus chemotherapy.

To investigate this heterogeneity, subgroup analyses were performed. The results suggested numerically greater pCR benefit in specific biomarker-defined populations: a significantly higher pCR rate was observed in the PD-L1 positive (CPS ≥1) subgroup (RR: 1.81, 95% CI: 1.02 to 3.19, *P* = 0.042) and the microsatellite instability (MSI) subgroup (RR: 2.50, 95% CI: 1.20 to 5.23, *P* = 0.015), with no heterogeneity detected in either subgroup (*I²* = 0.0%). Conversely, the improvement in pCR was not statistically significant in patients with PD-L1 negative (CPS <1; RR: 1.60, 95% CI: 0.78 to 3.29, *P* = 0.204) or microsatellite stable (MSS; RR: 1.89, 95% CI: 0.98 to 3.64, *P* = 0.058) tumors (**Figure 3**). However, the interaction tests were not statistically significant for either PD-L1 expression (*P* for interaction = 0.79) or MSI status (*P* for interaction = 0.58).

Furthermore, we performed sensitivity analyses excluding the Peng et al. trial (16), which enrolled HER2-positive patients and used a trastuzumab-containing backbone. The pCR benefit remained consistent (RR: 2.94, 95% CI: 2.12 to 4.09, *P* < 0.001; **Supplementary Figure 2**).

### 3.4 R0 resection rate

**Figure 4:**
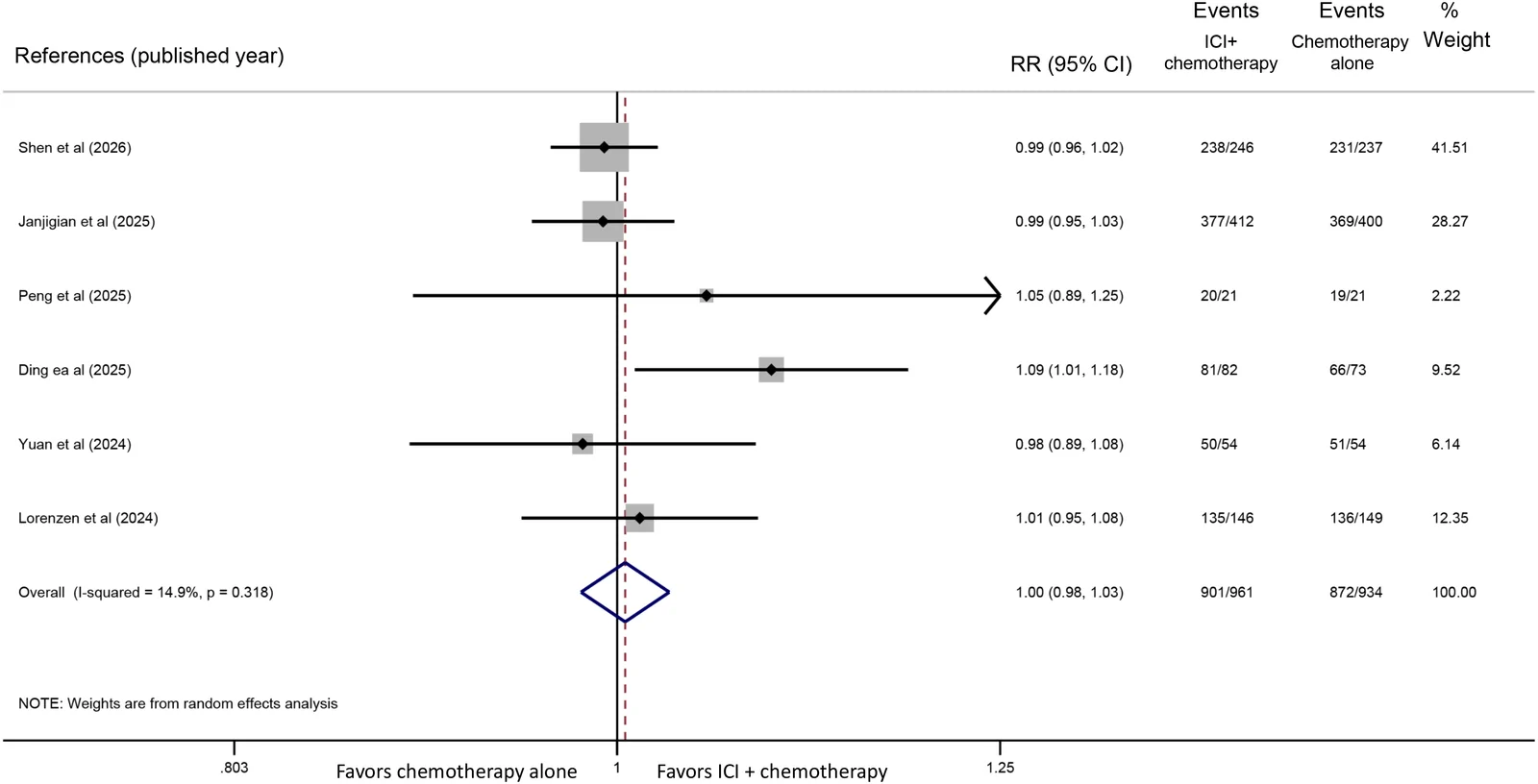
Forest plot of R0 resection rates comparing perioperative ICIs plus chemotherapy versus chemotherapy alone in patients with resectable gastric/gastroesophageal junction cancer. ICIs, immune checkpoint inhibitors. RR >1 favors ICIs plus chemotherapy.

Six studies provided data on the R0 resection rate (14, 16–18, 21, 22). The pooled analysis demonstrated that the addition of ICIs to chemotherapy did not significantly differ from chemotherapy alone with respect to the R0 resection rate (RR: 1.00, 95% CI: 0.98 to 1.03, *P* = 0.724), with low heterogeneity (*I²* = 14.9%) (**Figure 4**). While this finding indicates comparable rates of margin-negative resection between the two groups, it should be interpreted with caution, as R0 resection alone does not fully capture perioperative feasibility. Other metrics, such as surgical completion rate and treatment adherence, are also relevant for a comprehensive assessment.

### 3.5 Event-free survival and overall survival

**Figure 5:**
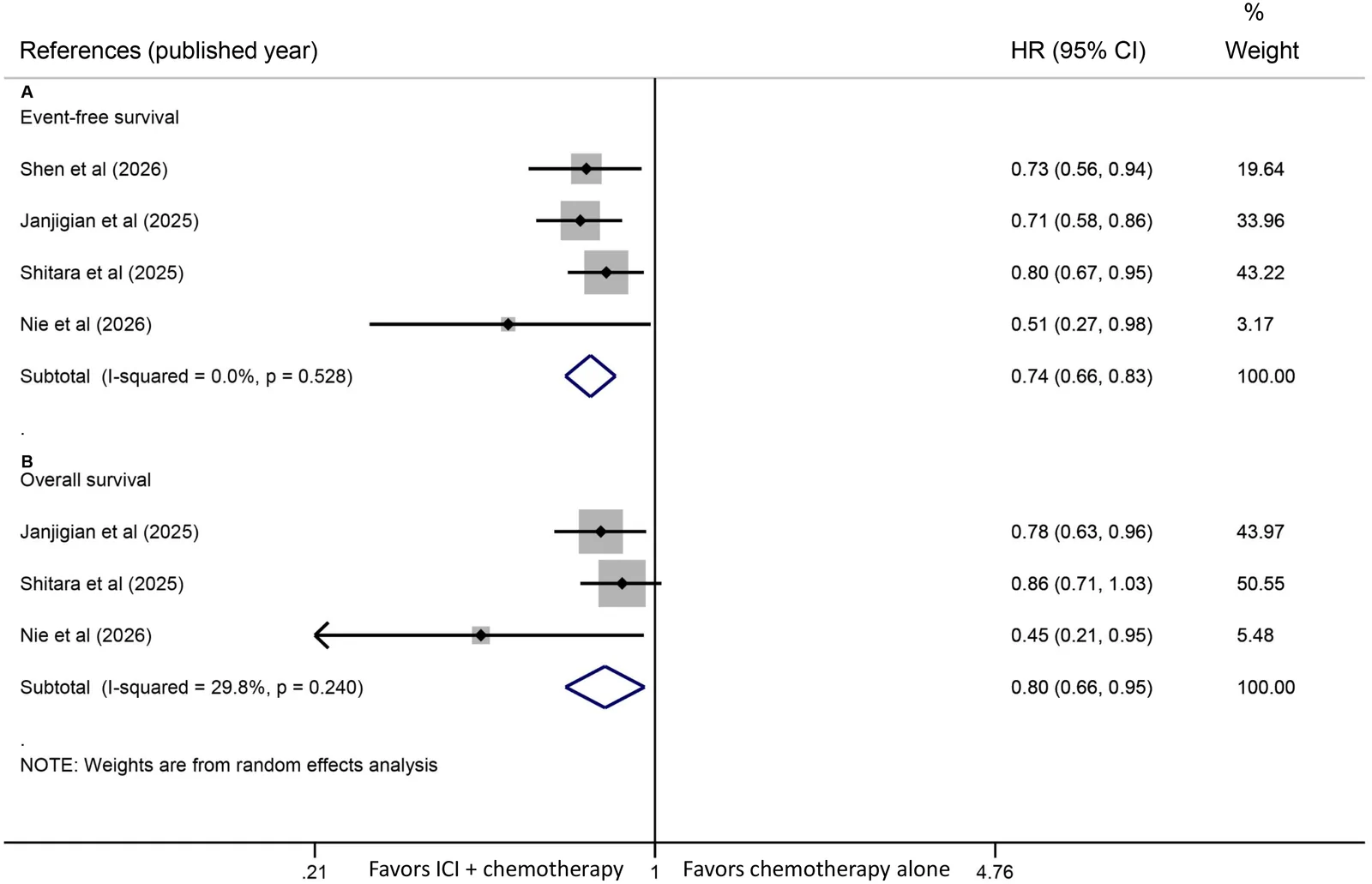
Forest plot of **(A)** event-free and **(B)** overall survival comparing perioperative ICIs plus chemotherapy versus chemotherapy alone in patients with resectable gastric/gastroesophageal junction cancer. ICIs, immune checkpoint inhibitors. HR <1 favors ICIs plus chemotherapy.

Pooled analysis demonstrated that the combination of ICI and chemotherapy significantly prolonged both EFS (HR: 0.74, 95% CI: 0.66 to 0.83, *P* < 0.001; *I²* = 0.0%, based on four trials (15, 18, 20, 21)) and OS (HR: 0.80, 95% CI: 0.66 to 0.95, *P* = 0.013; *I²* = 29.8%, based on three trials (15, 18, 20)) compared to chemotherapy alone (**Figure 5**). Subgroup meta-analysis of EFS demonstrated that the EFS benefit was more pronounced in patients with CPS ≥1 (HR: 0.74, 95% CI: 0.63 to 0.85, *P* < 0.001) than in those with CPS <1 (HR: 0.84, 95% CI: 0.61 to 1.17, *P* = 0.310) (**Supplementary Figure 3**).

### 3.6 Safety profile

**Figure 6:**
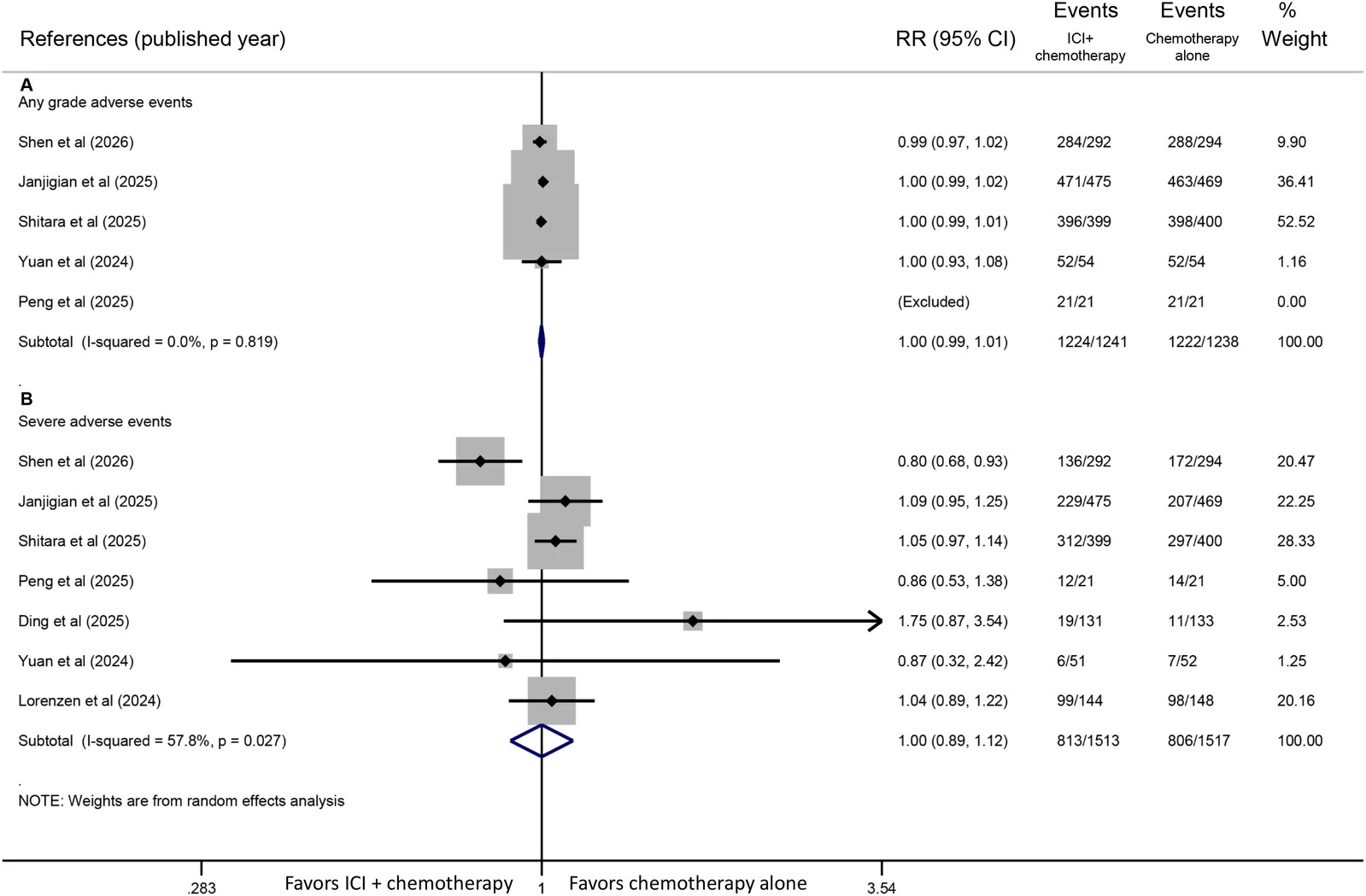
Comparative safety profiles of perioperative ICIs plus chemotherapy versus chemotherapy alone in patients with resectable gastric/gastroesophageal junction cancer, including **(A)** any grade and **(B)** severe adverse event. ICIs, immune checkpoint inhibitors. RR <1 favors ICIs plus chemotherapy.

Pooled analysis revealed that the combination of ICI and chemotherapy did not significantly increase the incidence of any-grade AEs compared to chemotherapy alone (RR: 1.00, 95% CI: 0.99 to 1.01, *P* = 0.916; *I²* = 0.0%), based on data from five studies (**Figure 6A**). Furthermore, analysis of severe AEs from all seven studies showed no statistically significant difference between the treatment arms (RR: 1.00, 95% CI: 0.89 to 1.12, *P* = 1.00; *I²* = 57.8%) (**Figure 6B**).

## 4 Discussion

A recently published meta-analysis has synthesized the evidence on perioperative immunochemotherapy for gastric/GEJ cancer (23). Nevertheless, several clinically relevant questions remain unanswered, and our study distinguishes itself from this prior work in several key aspects. First, in terms of scope and scale, our meta-analysis includes 7 RCTs encompassing 3252 patients, compared with 4 RCTs and 2358 patients in the previous analysis, providing a substantially larger evidence base for all key endpoints, including pCR, R0 resection, EFS, OS, and safety. Second, we have incorporated the most recent survival updates from pivotal trials that were not available in the prior analysis, including the final OS analysis of the MATTERHORN trial (presented at the 2025 ESMO Congress) (19) and the 3-year follow-up results of the NEOSUMMIT-01 (20) and ASTRUM-006 trials (21). Third, we have performed detailed biomarker-driven subgroup analyses stratified by standardized PD-L1 expression and MSI status, providing a more nuanced understanding of treatment effect modification.

Based on this updated evidence, our pooled analysis demonstrated that the addition of ICIs to chemotherapy significantly improved the pCR rate compared with chemotherapy alone. Importantly, the combination therapy also led to statistically significant prolongations of both EFS and OS. Notably, the pCR and EFS benefits were consistently observed in PD-L1-positive populations. Furthermore, the safety profile showed no statistically significant difference in any-grade or severe adverse events between the two groups.

The included RCTs used various chemotherapy regimens, primarily FLOT and XELOX/SOX, which are recommended by the National Comprehensive Cancer Network (NCCN) (24), the European Society for Medical Oncology (ESMO) (2), and the Chinese Society of Clinical Oncology (CSCO) (25) for locally advanced gastric/GEJ cancer. In the chemotherapy-alone groups, pCR rates ranged from 7% to 15% with FLOT and from 6% to 14% with XELOX/SOX. Consistent pCR benefits with the addition of ICIs were observed across these chemotherapy backbones, suggesting that the efficacy of ICI-chemotherapy combination is maintained regardless of the specific chemotherapy regimen or patient ethnicity.

Our analysis showed a pooled pCR rate of 19% in the ICI-chemotherapy group, compared with 7% in the chemotherapy-alone group. These findings align with a recent meta-analysis, which reported a pCR rate of 24% with neoadjuvant immunotherapy in resectable gastric/GEJ tumors, although that analysis primarily included single-arm studies (26). Moreover, the phase II trial NEOSUMMIT-03, evaluated the efficacy of neoadjuvant toripalimab plus chemotherapy in locally advanced GEJ cancer and reported a pCR rate of 28.1% with a manageable safety profile, further corroborating our findings (27).

However, the moderate heterogeneity (*I²* = 40.2%) observed across the included trials suggests some variability in the pCR benefit across studies, though the magnitude is modest. The heterogeneity may stem from several sources, including differences in ICI agents (PD-1 vs. PD-L1 inhibitors), chemotherapy backbones (FLOT vs. XELOX/SOX), and patient populations (e.g., PD-L1 expression and MSI status). Nevertheless, the *I²* value of 40.2% falls below the conventional threshold for substantial heterogeneity, suggesting that the observed variability is unlikely to undermine the overall conclusion. Given the limited number of trials and the residual clinical heterogeneity among them, the consistency of pCR benefit across different therapeutic regimens and patient subgroups merits further investigation in future studies.

Notably, a phase II trial (NEONIPIGA) evaluating neoadjuvant dual-ICI therapy (nivolumab plus ipilimumab) in a highly selected population of patients with deficient mismatch repair/MSI-H tumors reported a remarkably high pCR rate of 58.6% (9). In parallel, the phase III DRAGON IV/CAP 05 trial evaluated perioperative camrelizumab (anti-PD-1) plus rivoceranib (anti-VEGFR2) and chemotherapy versus chemotherapy alone in locally advanced gastric cancer and demonstrated a significant improvement in pCR rates with the triplet combination (18.3% vs. 5.0%) (28). Collectively, these findings highlight the potential of ICI-based combination strategies, whether with another ICI or with an anti-angiogenic agent, to enhance pathological responses. However, whether such approaches confer additional benefits beyond ICI–chemotherapy in biomarker-unselected or MSS populations warrants further investigation in large-scale RCTs.

Consistent with previous research indicating that pCR serves as a robust predictor of overall survival in patients receiving neoadjuvant immunotherapy (29), our meta-analysis suggests that the beneficial effect of ICIs on pathological responses translates into long-term survival gains in resectable gastric/GEJ cancer. It is noteworthy that the adjuvant use of ICIs plus chemotherapy has not demonstrated significant survival benefits in gastric/GEJ cancer (30). Therefore, the survival advantage observed in our analysis may be attributable to the perioperative integration of ICIs with chemotherapy, further supporting this treatment strategy.

PD-L1 expression is a recognized predictor of response to immunotherapy (7, 31). In our subgroup analyses, the pCR benefit appeared numerically greater in the PD-L1-positive subgroup (CPS ≥1), and this finding was supported by two included RCTs (15, 18), both of which reported better EFS outcomes in PD-L1-positive than in PD-L1-negative patients. However, the test for interaction between PD-L1-positive and PD-L1-negative subgroups was not statistically significant, indicating no formal evidence that PD-L1 expression modifies the treatment effect. Thus, the observed findings should be considered hypothesis-generating and warrant further investigation in well-powered prospective studies.

Several limitations of this meta-analysis should be acknowledged. First, the inclusion of only seven RCTs, one of which had a relatively small sample size (42 patients), limits the statistical power of our analysis, particularly in subgroup assessments. Moreover, the limited number of included trials precluded more robust meta-regression or stratified analyses to fully account for clinical heterogeneity arising from differences in ICI agents, chemotherapy backbones, and patient populations. While we performed subgroup and sensitivity analyses to explore the impact of these variables on the pooled estimates, the small number of studies limits our ability to draw definitive conclusions about effect modification by these clinical factors. Consequently, findings from subgroup analyses, such as those based on PD-L1 expression or microsatellite status, should be interpreted as exploratory rather than definitive. Second, while we assessed the R0 resection rate as a secondary outcome, other important measures of perioperative feasibility, such as surgical completion rate, reasons for non-completion, and preoperative/postoperative treatment adherence, were not consistently reported across trials and could not be pooled. Therefore, the R0 finding should not be overinterpreted as a complete assessment of perioperative feasibility; dedicated analyses with more granular surgical data are warranted. Third, while the survival benefit was consistent across the included trials with low statistical heterogeneity, the evidence is primarily derived from a limited number of trials (four studies), and the follow-up durations for some trials remain relatively short. The generalizability of the survival benefit to other ICI regimens, chemotherapy backbones, and patient populations beyond those studied requires further validation in well-powered prospective studies with extended follow-up. Finally, PD-L1 testing methods and cutoff definitions varied across trials. To ensure comparability, we restricted the PD-L1 subgroup meta-analysis to studies reporting CPS ≥1 versus CPS <1 data; trials using alternative cutoffs were excluded. Consequently, this subgroup analysis was based on a limited number of studies, and the findings should be interpreted as exploratory rather than definitive. Future studies with harmonized PD-L1 assessment methods and larger sample sizes are needed to more reliably identify patient subgroups most likely to benefit from perioperative immunochemotherapy.

In summary, this meta-analysis provides updated evidence that the addition of ICIs to perioperative chemotherapy significantly improves pCR rates and confers meaningful survival benefits in patients with resectable gastric or GEJ cancer. These findings further reinforce the use of ICIs combined with chemotherapy as an increasingly promising perioperative treatment option for this patient population. However, given the limited number of trials, the heterogeneity in treatment regimens, and the lack of long-term follow-up data for some studies, these results should be interpreted with appropriate caution. Confirmation in larger, well-designed trials with extended follow-up is needed before this approach can be universally recommended as a new standard of care.

## Data Availability

The original contributions presented in the study are included in the article/**Supplementary Material**. Further inquiries can be directed to the corresponding authors.
