# Supplementary material for: Perioperative immunochemotherapy for resectable gastric or gastroesophageal junction cancer: a meta-analysis of efficacy and safety from randomized trials
Source: Front Immunol. 2026 Aug 25;17:1765780. doi: 10.3389/fimmu.2026.1765780 (PMC13550002; doi:10.3389/fimmu.2026.1765780)
Supplement: Supplementary file 1 [file Table1.docx]

**Table S1. Search strategy for PubMed (inception to Nov 17, 2025)**

| **No.** | **Query Results** | **Results** |
| --- | --- | --- |
| #37 | #28 and #33 and #36 | 298 |
| #36 | #34 or #35 | 1,946,119 |
| #35 | Search: randomize*[Text Word] | 1,944,299 |
| #34 | Search: "Randomized Controlled Trial" [Publication Type] OR "Randomized Controlled Trials as Topic"[Mesh] | 835,958 |
| #33 | #29 or #30 or #31 or #32 | 380,302 |
| #32 | Search: "Stomach"[Mesh] | 134,280 |
| #31 | Search: "Esophagogastric Junction"[Mesh] | 11,128 |
| #30 | Search: gastroesophageal junction[Title/Abstract] | 4,287 |
| #29 | Search: gastric[Title/Abstract] | 319,966 |
| #28 | #1 or #2 or #3 or #4 or #5 or #6 or #7 or #8 or #9 or #10 or #11 or #12 or #13 or #14 or #15 or #16 or #17 or #18 or #19 or #20 or #21 or #22 or #23 or #24 or #25 or #26 or #27 | 85,002 |
| #27 | Search: "CTLA-4 Antigen"[Mesh] | 7,614 |
| #26 | Search: "Programmed Cell Death 1 Receptor"[Mesh] | 14,793 |
| #25 | Search: "Immune Checkpoint Inhibitors"[Mesh] | 17,169 |
| #24 | Search: Sugemalimab*[Title/Abstract] | 52 |
| #23 | Search: Envafolimab*[Title/Abstract] | 41 |
| #22 | Search: Penpulimab*[Title/Abstract] | 45 |
| #21 | Search: Toripalimab*[Title/Abstract] | 518 |
| #20 | Search: Camrelizumab*[Title/Abstract] | 964 |
| #19 | Search: Sintilimab*[Title/Abstract] | 907 |
| #18 | Search: Tislelizumab*[Title/Abstract] | 708 |
| #17 | Search: ipilimumab*[Title/Abstract] | 6,136 |
| #16 | Search: dostarlimab*[Title/Abstract] | 232 |
| #15 | Search: cemiplimab*[Title/Abstract] | 641 |
| #14 | Search: tremelimumab*[Title/Abstract] | 739 |
| #13 | Search: avelumab*[Title/Abstract] | 1,289 |
| #12 | Search: durvalumab*[Title/Abstract] | 2,507 |
| #11 | Search: atezolizumab*[Title/Abstract] | 4,463 |
| #10 | Search: nivolumab*[Title/Abstract] | 11,910 |
| #9 | Search: pembrolizumab*[Title/Abstract] | 12,148 |
| #8 | Search: PD-L1 antibod*[Title/Abstract] | 2,237 |
| #7 | Search: PD-1 antibod*[Title/Abstract] | 3,253 |
| #6 | Search: PD-L1 inhibitor*[Title/Abstract] | 3,152 |
| #5 | Search: PD-1 inhibitor*[Title/Abstract] | 4,263 |
| #4 | Search: CTLA-4 antigen[Title/Abstract] | 99 |
| #3 | Search: CTLA-4 inhibitor*[Title/Abstract] | 624 |
| #2 | Search: ICIs[Title/Abstract] | 12,727 |
| #1 | Search: checkpoint inhibitor*[Title/Abstract] | 44,765 |

**Table S2. Search strategy for PubMed (Nov 18, 2025 to Jun 30, 2026)**

| **No.** | **Query Results** | **Results** |
| --- | --- | --- |
| #38 | Search: #29 and #34 and #37 Filters: from 2025/11/18 - 2026/6/30 | 66 |
| #37 | Search: #35 or #36 Filters: from 2025/11/18 - 2026/6/30 | 56,328 |
| #36 | Search: randomize*[Text Word] Filters: from 2025/11/18 - 2026/6/30 | 56,248 |
| #35 | Search: "Randomized Controlled Trial" [Publication Type] OR "Randomized Controlled Trials as Topic"[Mesh] Filters: from 2025/11/18 - 2026/6/30 | 25,873 |
| #34 | Search: #30 or #31 or #32 or #33 Filters: from 2025/11/18 - 2026/6/30 | 11,027 |
| #33 | Search: "Stomach"[Mesh] Filters: from 2025/11/18 - 2026/6/30 | 948 |
| #32 | Search: "Esophagogastric Junction"[Mesh] Filters: from 2025/11/18 - 2026/6/30 | 239 |
| #31 | Search: gastroesophageal junction[Title/Abstract] Filters: from 2025/11/18 - 2026/6/30 | 349 |
| #30 | Search: gastric[Title/Abstract] Filters: from 2025/11/18 - 2026/6/30 | 10,746 |
| #29 | Search: #1 or #2 or #3 or #4 or #5 or #6 or #7 or #8 or #9 or #10 or #11 or #12 or #13 or #14 or #15 or #16 or #17 or #18 or #19 or #20 or #21 or #22 or #23 or #24 or #25 or #26 or #27 or #28 Filters: from 2025/11/18 - 2026/6/30 | 23,325 |
| #28 | Search: "CTLA-4 Antigen"[Mesh] Filters: from 2025/11/18 - 2026/6/30 | 131 |
| #27 | Search: "Programmed Cell Death 1 Receptor"[Mesh] Filters: from 2025/11/18 - 2026/6/30 | 915 |
| #26 | Search: "Immune Checkpoint Inhibitors"[Mesh] Filters: from 2025/11/18 - 2026/6/30 | 2,949 |
| #25 | Search: immunotherapy[Title/Abstract] Filters: from 2025/11/18 - 2026/6/30 | 18,168 |
| #24 | Search: Sugemalimab*[Title/Abstract] Filters: from 2025/11/18 - 2026/6/30 | 15 |
| #23 | Search: Envafolimab*[Title/Abstract] Filters: from 2025/11/18 - 2026/6/30 | 24 |
| #22 | Search: Penpulimab*[Title/Abstract] Filters: from 2025/11/18 - 2026/6/30 | 28 |
| #21 | Search: Toripalimab*[Title/Abstract] Filters: from 2025/11/18 - 2026/6/30 | 127 |
| #20 | Search: Camrelizumab*[Title/Abstract] Filters: from 2025/11/18 - 2026/6/30 | 162 |
| #19 | Search: Sintilimab*[Title/Abstract] Filters: from 2025/11/18 - 2026/6/30 | 202 |
| #18 | Search: Tislelizumab*[Title/Abstract] Filters: from 2025/11/18 - 2026/6/30 | 231 |
| #17 | Search: ipilimumab*[Title/Abstract] Filters: from 2025/11/18 - 2026/6/30 | 486 |
| #16 | Search: dostarlimab*[Title/Abstract] Filters: from 2025/11/18 - 2026/6/30 | 45 |
| #15 | Search: cemiplimab*[Title/Abstract] Filters: from 2025/11/18 - 2026/6/30 | 112 |
| #14 | Search: tremelimumab*[Title/Abstract] Filters: from 2025/11/18 - 2026/6/30 | 115 |
| #13 | Search: avelumab*[Title/Abstract] Filters: from 2025/11/18 - 2026/6/30 | 135 |
| #12 | Search: durvalumab*[Title/Abstract] Filters: from 2025/11/18 - 2026/6/30 | 456 |
| #11 | Search: atezolizumab*[Title/Abstract] Filters: from 2025/11/18 - 2026/6/30 | 603 |
| #10 | Search: nivolumab*[Title/Abstract] Filters: from 2025/11/18 - 2026/6/30 | 1,173 |
| #9 | Search: pembrolizumab*[Title/Abstract] Filters: from 2025/11/18 - 2026/6/30 | 1,588 |
| #8 | Search: PD-L1 antibod*[Title/Abstract] Filters: from 2025/11/18 - 2026/6/30 | 208 |
| #7 | Search: PD-1 antibod*[Title/Abstract] Filters: from 2025/11/18 - 2026/6/30 | 269 |
| #6 | Search: PD-L1 inhibitor*[Title/Abstract] Filters: from 2025/11/18 - 2026/6/30 | 413 |
| #5 | Search: PD-1 inhibitor*[Title/Abstract] Filters: from 2025/11/18 - 2026/6/30 | 665 |
| #4 | Search: CTLA-4 antigen[Title/Abstract] Filters: from 2025/11/18 - 2026/6/30 | 3 |
| #3 | Search: CTLA-4 inhibitor*[Title/Abstract] Filters: from 2025/11/18 - 2026/6/30 | 72 |
| #2 | Search: ICIs[Title/Abstract] Filters: from 2025/11/18 - 2026/6/30 | 2,550 |
| #1 | Search: checkpoint inhibitor*[Title/Abstract] Filters: from 2025/11/18 - 2026/6/30 | 6,750 |

**Table S3. Search strategy for Embase (inception to Nov 17, 2025)**

| **No.** | **Query Results** | **Results** |
| --- | --- | --- |
| #37 | #28 and #33 and #36 | 1,011 |
| #36 | #34 or #35 | 2,693,074 |
| #35 | 'randomized controlled trial'/exp | 1,127,836 |
| #34 | random*:ti,ab,kw | 2,547,127 |
| #33 | #29 or #30 or #31 or #32 | 574,965 |
| #32 | 'stomach'/exp | 222 |
| #31 | 'gastroesophageal junction'/exp | 12,646 |
| #30 | 'gastroesophageal junction':ti,ab,kw | 9,444 |
| #29 | 'gastric':ti,ab,kw | 462,851 |
| #28 | #1 or #2 or #3 or #4 or #5 or #6 or #7 or #8 or #9 or #10 or #11 or #12 or #13 or #14 or #15 or #16 or #17 or #18 or #19 or #20 or #21 or #22 or #23 or #24 or #25 or #26 or #27 | 198,674 |
| #27 | 'cytotoxic t lymphocyte antigen 4'/exp | 38,800 |
| #26 | 'programmed death 1 receptor'/exp | 53,081 |
| #25 | 'immune checkpoint inhibitor'/exp | 50,497 |
| #24 | 'sugemalimab*':ti,ab,kw | 87 |
| #23 | 'envafolimab*':ti,ab,kw | 187 |
| #22 | 'penpulimab*':ti,ab,kw | 214 |
| #21 | 'toripalimab*':ti,ab,kw | 1,541 |
| #20 | 'camrelizumab*':ti,ab,kw | 2,055 |
| #19 | 'sintilimab*':ti,ab,kw | 2,101 |
| #18 | 'tislelizumab*':ti,ab,kw | 2,198 |
| #17 | 'ipilimumab*':ti,ab,kw | 14,760 |
| #16 | 'dostarlimab*':ti,ab,kw | 610 |
| #15 | 'cemiplimab*':ti,ab,kw | 1,528 |
| #14 | 'tremelimumab*':ti,ab,kw | 1,929 |
| #13 | 'avelumab*':ti,ab,kw | 3,178 |
| #12 | 'durvalumab*':ti,ab,kw | 7,046 |
| #11 | 'atezolizumab*':ti,ab,kw | 10,757 |
| #10 | 'nivolumab*':ti,ab,kw | 28,338 |
| #9 | 'pembrolizumab*':ti,ab,kw | 29,988 |
| #8 | 'pd-l1 antibod*':ti,ab,kw | 4,657 |
| #7 | 'pd-1 antibod*':ti,ab,kw | 7,593 |
| #6 | 'pd-l1 inhibitor*':ti,ab,kw | 5,808 |
| #5 | 'pd-1 inhibitor*':ti,ab,kw | 8,692 |
| #4 | 'ctla-4 antigen':ti,ab,kw | 124 |
| #3 | 'ctla-4 inhibitor*':ti,ab,kw | 1,196 |
| #2 | 'icis':ti,ab,kw | 19,574 |
| #1 | 'checkpoint inhibitor*':ti,ab,kw | 74,551 |

**Table S4. Search strategy for Embase (Nov 18, 2025 to Jun 30, 2026)**

| **No.** | **Query Results** | **Results** |
| --- | --- | --- |
| #37 | #28 and #33 and #36 | 288 |
| #36 | #34 or #35 | 175,246 |
| #35 | 'randomized controlled trial'/exp | 98,426 |
| #34 | random*:ti,ab,kw | 157,757 |
| #33 | #29 or #30 or #31 or #32 | 28,320 |
| #32 | 'stomach'/exp | 22,816 |
| #31 | 'gastroesophageal junction'/exp | 1,829 |
| #30 | 'gastroesophageal junction':ti,ab,kw | 1,057 |
| #29 | 'gastric':ti,ab,kw | 19,574 |
| #28 | #1 or #2 or #3 or #4 or #5 or #6 or #7 or #8 or #9 or #10 or #11 or #12 or #13 or #14 or #15 or #16 or #17 or #18 or #19 or #20 or #21 or #22 or #23 or #24 or #25 or #26 or #27 | 24,905 |
| #27 | 'cytotoxic t lymphocyte antigen 4'/exp | 2,774 |
| #26 | 'programmed death 1 receptor'/exp | 2,474 |
| #25 | 'immune checkpoint inhibitor'/exp | 11,906 |
| #24 | 'sugemalimab*':ti,ab,kw | 22 |
| #23 | 'envafolimab*':ti,ab,kw | 69 |
| #22 | 'penpulimab*':ti,ab,kw | 61 |
| #21 | 'toripalimab*':ti,ab,kw | 383 |
| #20 | 'camrelizumab*':ti,ab,kw | 384 |
| #19 | 'sintilimab*':ti,ab,kw | 481 |
| #18 | 'tislelizumab*':ti,ab,kw | 676 |
| #17 | 'ipilimumab*':ti,ab,kw | 1,333 |
| #16 | 'dostarlimab*':ti,ab,kw | 162 |
| #15 | 'cemiplimab*':ti,ab,kw | 342 |
| #14 | 'tremelimumab*':ti,ab,kw | 286 |
| #13 | 'avelumab*':ti,ab,kw | 383 |
| #12 | 'durvalumab*':ti,ab,kw | 1,269 |
| #11 | 'atezolizumab*':ti,ab,kw | 1,590 |
| #10 | 'nivolumab*':ti,ab,kw | 3,093 |
| #9 | 'pembrolizumab*':ti,ab,kw | 4,632 |
| #8 | 'pd-l1 antibod*':ti,ab,kw | 384 |
| #7 | 'pd-1 antibod*':ti,ab,kw | 661 |
| #6 | 'pd-l1 inhibitor*':ti,ab,kw | 800 |
| #5 | 'pd-1 inhibitor*':ti,ab,kw | 1,491 |
| #4 | 'ctla-4 antigen':ti,ab,kw | 4 |
| #3 | 'ctla-4 inhibitor*':ti,ab,kw | 170 |
| #2 | 'icis':ti,ab,kw | 4,454 |
| #1 | 'checkpoint inhibitor*':ti,ab,kw | 11,625 |

**Table S5. Search strategy for the Cochrane Library (inception to Nov 2025)**

| **No.** | **Query Results** | **Results** |
| --- | --- | --- |
| #1 | (checkpoint inhibitor*):ti,ab,kw | 3,471 |
| #2 | (ICI*):ti,ab,kw | 3,129 |
| #3 | (CTLA-4 inhibitor*):ti,ab,kw | 408 |
| #4 | (CTLA-4 antigen):ti,ab,kw | 285 |
| #5 | (PD-1 inhibitor*):ti,ab,kw | 2,253 |
| #6 | (PD-L1 inhibitor*):ti,ab,kw | 2,006 |
| #7 | (PD-1 antibod*):ti,ab,kw | 2,026 |
| #8 | (PD-L1 antibod*):ti,ab,kw | 2,008 |
| #9 | (pembrolizumab*):ti,ab,kw | 4,159 |
| #10 | (nivolumab*):ti,ab,kw | 3,674 |
| #11 | (atezolizumab*):ti,ab,kw | 1,790 |
| #12 | (durvalumab*):ti,ab,kw | 1,554 |
| #13 | (avelumab*):ti,ab,kw | 514 |
| #14 | (tremelimumab*):ti,ab,kw | 529 |
| #15 | (cemiplimab*):ti,ab,kw | 230 |
| #16 | (dostarlimab*):ti,ab,kw | 181 |
| #17 | (ipilimumab*):ti,ab,kw | 2,181 |
| #18 | (Tislelizumab*):ti,ab,kw | 428 |
| #19 | (Sintilimab*):ti,ab,kw | 325 |
| #20 | (Camrelizumab*):ti,ab,kw | 374 |
| #21 | (Toripalimab*):ti,ab,kw | 275 |
| #22 | (Penpulimab*):ti,ab,kw | 51 |
| #23 | (Envafolimab*):ti,ab,kw | 29 |
| #24 | (Sugemalimab*):ti,ab,kw | 28 |
| #25 | MeSH descriptor: [Immune Checkpoint Inhibitors] explode all trees | 414 |
| #26 | MeSH descriptor: [Programmed Cell Death 1 Receptor] explode all trees | 251 |
| #27 | MeSH descriptor: [CTLA-4 Antigen] explode all trees | 109 |
| #28 | #1 or #2 or #3 or #4 or #5 or #6 or #7 or #8 or #9 or #10 or #11 or #12 or #13 or #14 or #15 or #16 or #17 or #18 or #19 or #20 or #21 or #22 or #23 or #24 or #25 or #26 or #27 | 17,490 |
| #29 | (gastric):ti,ab,kw | 33,524 |
| #30 | (gastroesophageal junction):ti,ab,kw | 1,877 |
| #31 | MeSH descriptor: [Esophagogastric Junction] explode all trees | 744 |
| #32 | MeSH descriptor: [Stomach] explode all trees | 4,193 |
| #33 | #29 or #30 or #31 or #32 | 34,706 |
| #34 | (random*):ti,ab,kw | 1,437,787 |
| #35 | MeSH descriptor: [Randomized Controlled Trial] explode all trees | 34 |
| #36 | #34 or #35 | 1,437,787 |
| #37 | #28 and #33 and #36 | 611 |

**Table S6. Search strategy for the Cochrane Library (****Nov 2025 to Jun 2026)**

| **No.** | **Query Results** | **Results** |
| --- | --- | --- |
| #1 | (checkpoint inhibitor*):ti,ab,kw | 547 |
| #2 | (ICI*):ti,ab,kw | 429 |
| #3 | (CTLA-4 inhibitor*):ti,ab,kw | 46 |
| #4 | (CTLA-4 antigen):ti,ab,kw | 15 |
| #5 | (PD-1 inhibitor*):ti,ab,kw | 361 |
| #6 | (PD-L1 inhibitor*):ti,ab,kw | 300 |
| #7 | (PD-1 antibod*):ti,ab,kw | 261 |
| #8 | (PD-L1 antibod*):ti,ab,kw | 244 |
| #9 | (pembrolizumab*):ti,ab,kw | 611 |
| #10 | (nivolumab*):ti,ab,kw | 402 |
| #11 | (atezolizumab*):ti,ab,kw | 201 |
| #12 | (durvalumab*):ti,ab,kw | 205 |
| #13 | (avelumab*):ti,ab,kw | 68 |
| #14 | (tremelimumab*):ti,ab,kw | 58 |
| #15 | (cemiplimab*):ti,ab,kw | 56 |
| #16 | (dostarlimab*):ti,ab,kw | 39 |
| #17 | (ipilimumab*):ti,ab,kw | 182 |
| #18 | (Tislelizumab*):ti,ab,kw | 142 |
| #19 | (Sintilimab*):ti,ab,kw | 91 |
| #20 | (Camrelizumab*):ti,ab,kw | 74 |
| #21 | (Toripalimab*):ti,ab,kw | 89 |
| #22 | (Penpulimab*):ti,ab,kw | 18 |
| #23 | (Envafolimab*):ti,ab,kw | 11 |
| #24 | (Sugemalimab*):ti,ab,kw | 2 |
| #25 | MeSH descriptor: [Immune Checkpoint Inhibitors] explode all trees | 81 |
| #26 | MeSH descriptor: [Programmed Cell Death 1 Receptor] explode all trees | 29 |
| #27 | MeSH descriptor: [CTLA-4 Antigen] explode all trees | 20 |
| #28 | #1 or #2 or #3 or #4 or #5 or #6 or #7 or #8 or #9 or #10 or #11 or #12 or #13 or #14 or #15 or #16 or #17 or #18 or #19 or #20 or #21 or #22 or #23 or #24 or #25 or #26 or #27 | 2,398 |
| #29 | (gastric):ti,ab,kw | 1,410 |
| #30 | (gastroesophageal junction):ti,ab,kw | 208 |
| #31 | MeSH descriptor: [Esophagogastric Junction] explode all trees | 22 |
| #32 | MeSH descriptor: [Stomach] explode all trees | 11 |
| #33 | #29 or #30 or #31 or #32 | 1,438 |
| #34 | (random*):ti,ab,kw | 81,058 |
| #35 | MeSH descriptor: [Randomized Controlled Trial] explode all trees | 34 |
| #36 | #34 or #35 | 81,058 |
| #37 | #28 and #33 and #36 | 118 |

**Table S7. Characteristics of excluded full-text studies (inception to Nov 17, 2025)**

| **Study** | **Reason for exclusion** |
| --- | --- |
| Lin et al ^(1)^ | Other comparison: camrelizumab + apatinib + chemotherapy vs. chemotherapy |
| Li et al ^(2)^ | Other comparison: camrelizumab + rivoceranib + chemotherapy vs. chemotherapy |
| Lordick et al ^(3)^ | Not perioperative: adjuvant Nivolumab/ipilimumab vs. chemotherapy |
| Kang et al ^(4)^ | Not perioperative: adjuvant Nivolumab + chemotherapy vs. chemotherapy |
| Lin et al ^(5)^ | Not perioperative: neoadjuvant camrelizumab + apatinib + chemotherapy vs. chemotherapy |
| Janjigian et al ^(6)^ | Not resectable gastric/gastroesophageal junction cancer |
| Janjigian et al ^(7)^ | Not resectable gastric/gastroesophageal junction cancer |
| Qiu et al ^(8)^ | Not resectable gastric/gastroesophageal junction cancer |
| Rha et al ^(9)^ | Not resectable gastric/gastroesophageal junction cancer |
| Shen et al ^(10)^ | Not resectable gastric/gastroesophageal junction cancer |
| Zhang et al ^(11)^ | Not resectable gastric/gastroesophageal junction cancer |
| Cruz-Correa et al ^(12)^ | Not resectable gastric/gastroesophageal junction cancer |
| Alsina et al ^(13)^ | Not RCT |
| Shitara et al ^(14)^ | Other reasons: interim analysis of the included RCT |

Abbreviations: RCT: randomized controlled trial.

**References**

1.	Lin JX, Huang ZN, Tang YH, Ye K, Wang ZF, Cai LS, et al. Perioperative camrelizumab and apatinib combined with chemotherapy for locally advanced gastric cancer in China: three-year survival outcomes of phase 2 randomized controlled trial. EClinicalMedicine (2025) 89:103568. doi:10.1016/j.eclinm.2025.103568

2.	Li C, Tian Y, Zheng Y, Yuan F, Shi Z, Yang L, et al. Pathologic Response of Phase III Study: Perioperative Camrelizumab Plus Rivoceranib and Chemotherapy Versus Chemotherapy for Locally Advanced Gastric Cancer (DRAGON IV/CAP 05). J Clin Oncol (2025) 43(4):464-74. doi:10.1200/jco.24.00795

3.	Lordick F, Mauer ME, Stocker G, Cella CA, Ben-Aharon I, Piessen G, et al. Adjuvant immunotherapy in patients with resected gastric and oesophagogastric junction cancer following preoperative chemotherapy with high risk for recurrence (ypN+ and/or R1): European Organisation of Research and Treatment of Cancer (EORTC) 1707 VESTIGE study. Ann Oncol (2025) 36(2):197-207. doi:10.1016/j.annonc.2024.10.829

4.	Kang YK, Terashima M, Kim YW, Boku N, Chung HC, Chen JS, et al. Adjuvant nivolumab plus chemotherapy versus placebo plus chemotherapy for stage III gastric or gastro-oesophageal junction cancer after gastrectomy with D2 or more extensive lymph-node dissection (ATTRACTION-5): a randomised, multicentre, double-blind, placebo-controlled, phase 3 trial. Lancet Gastroenterol Hepatol (2024) 9(8):705-17. doi:10.1016/s2468-1253(24)00156-0

5.	Lin JX, Tang YH, Zheng HL, Ye K, Cai JC, Cai LS, et al. Neoadjuvant camrelizumab and apatinib combined with chemotherapy versus chemotherapy alone for locally advanced gastric cancer: a multicenter randomized phase 2 trial. Nat Commun (2024) 15(1):41. doi:10.1038/s41467-023-44309-5

6.	Janjigian YY, Shitara K, Moehler M, Garrido M, Salman P, Shen L, et al. First-line nivolumab plus chemotherapy versus chemotherapy alone for advanced gastric, gastro-oesophageal junction, and oesophageal adenocarcinoma (CheckMate 649): a randomised, open-label, phase 3 trial. Lancet (2021) 398(10294):27-40. doi:10.1016/s0140-6736(21)00797-2

7.	Janjigian YY, Kawazoe A, Bai Y, Xu J, Lonardi S, Metges JP, et al. Pembrolizumab plus trastuzumab and chemotherapy for HER2-positive gastric or gastro-oesophageal junction adenocarcinoma: interim analyses from the phase 3 KEYNOTE-811 randomised placebo-controlled trial. Lancet (2023) 402(10418):2197-208. doi:10.1016/s0140-6736(23)02033-0

8.	Qiu MZ, Oh DY, Kato K, Arkenau T, Tabernero J, Correa MC, et al. Tislelizumab plus chemotherapy versus placebo plus chemotherapy as first line treatment for advanced gastric or gastro-oesophageal junction adenocarcinoma: RATIONALE-305 randomised, double blind, phase 3 trial. Bmj (2024) 385:e078876. doi:10.1136/bmj-2023-078876

9.	Rha SY, Oh DY, Yañez P, Bai Y, Ryu MH, Lee J, et al. Pembrolizumab plus chemotherapy versus placebo plus chemotherapy for HER2-negative advanced gastric cancer (KEYNOTE-859): a multicentre, randomised, double-blind, phase 3 trial. Lancet Oncol (2023) 24(11):1181-95. doi:10.1016/s1470-2045(23)00515-6

10.	Shen L, Zhang Y, Li Z, Zhang X, Gao X, Liu B, et al. First-line cadonilimab plus chemotherapy in HER2-negative advanced gastric or gastroesophageal junction adenocarcinoma: a randomized, double-blind, phase 3 trial. Nat Med (2025) 31(4):1163-70. doi:10.1038/s41591-024-03450-4

11.	Zhang X, Wang J, Wang G, Zhang Y, Fan Q, Lu C, et al. First-Line Sugemalimab Plus Chemotherapy for Advanced Gastric Cancer: The GEMSTONE-303 Randomized Clinical Trial. Jama (2025) 333(15):1305-14. doi:10.1001/jama.2024.28463

12.	Cruz-Correa M, Oh DY, Kato K, Tabernero J, Bai Y, Shi J, et al. Tislelizumab + Chemotherapy in Gastric Cancer: Long-Term RATIONALE-305 Randomized Trial Follow-up. Adv Ther (2025). doi:10.1007/s12325-025-03415-0

13.	Alsina M, Villacampa G, de Andrea C, Vivancos A, Ponz-Sarvise M, Arrazubi V, et al. A Phase II Study of Perioperative Avelumab plus Chemotherapy for Patients with Resectable Gastric Cancer or Gastroesophageal Junction Cancer - The MONEO Study. Clin Cancer Res (2025) 31(14):2890-8. doi: 10.1158/1078-0432.Ccr-25-0369

14.	Shitara K, Rha SY, Wyrwicz LS, Oshima T, Karaseva N, Osipov M, et al. Neoadjuvant and adjuvant pembrolizumab plus chemotherapy in locally advanced gastric or gastro-oesophageal cancer (KEYNOTE-585): an interim analysis of the multicentre, double-blind, randomised phase 3 study. Lancet Oncol (2024) 25(2):212-24. doi: 10.1016/s1470-2045(23)00541-7

**Table S8. Characteristics of excluded full-text studies (Nov 18, 2025 to Jun 30, 2026)**

| **Study** | **Reason for exclusion** |
| --- | --- |
| Shitara et al ^(1)^ | Other comparison: zanidatamab + tislelizumab+ chemotherapy vs. zanidatamab + chemotherapy vs. trastuzumab + chemotherapy |
| Cruz-Correa et al ^(2)^ | Not resectable gastric/gastroesophageal junction cancer |
| Janjigian et al ^(3)^ | Not resectable gastric/gastroesophageal junction cancer |
| Janjigian et al ^(4)^ | Not resectable gastric/gastroesophageal junction cancer |
| Lowery et al ^(5)^ | Not resectable gastric/gastroesophageal junction cancer |
| Metges et al ^(6)^ | Not resectable gastric/gastroesophageal junction cancer |
| Peng et al ^(7)^ | Not resectable gastric/gastroesophageal junction cancer |
| Stein et al ^(8)^ | Not RCT |
| Zhang et al ^(9)^ | Not RCT |
| Zhou et al ^(10)^ | Not RCT |

**References**

1. Shitara K, Elimova E, Liu T, Tabernero J, Lee KW, Schenker M, et al. Zanidatamab with and without Tislelizumab in HER2-Positive Gastroesophageal Cancer. N Engl J Med (2026) 394(20):2002-14. doi: 10.1056/NEJMoa2517729

2. Cruz-Correa M, Oh DY, Kato K, Tabernero J, Bai Y, Shi J, et al. Tislelizumab + Chemotherapy in Gastric Cancer: Long-Term RATIONALE-305 Randomized Trial Follow-up. Adv Ther (2026) 43(1):165-83. doi: 10.1007/s12325-025-03415-0

3. Janjigian YY, Shitara K, Ajani JA, Shen L, Garrido M, Gallardo C, et al. Nivolumab plus chemotherapy as first-line treatment for advanced gastric, gastroesophageal junction, and esophageal adenocarcinoma: 5-year follow-up results from CheckMate 649. Ann Oncol (2026) 37(6):787-97. doi: 10.1016/j.annonc.2026.02.003

4. Janjigian YY, Smyth E, Shen L, Lee J, Hoff PM, Lonardi S, et al. DESTINY-Gastric05 phase III trial of first-line trastuzumab deruxtecan, chemotherapy, and pembrolizumab in HER2-positive gastric or gastroesophageal junction cancer. ESMO Gastrointest Oncol (2026) 11:100294. doi: 10.1016/j.esmogo.2025.100294

5. Lowery MA, Wyrwicz LS, Oh DY, Shiu KK, Yañez P, Bai Y, et al. First-line pembrolizumab plus chemotherapy for advanced HER2-negative gastric or gastroesophageal junction adenocarcinoma: Health-related quality of life from the randomized phase III KEYNOTE-859 trial. Eur J Cancer (2025) 231:115807. doi: 10.1016/j.ejca.2025.115807

6. Metges JP, Kato K, Sun JM, Shen L, Enzinger PC, Adenis A, et al. Pembrolizumab plus chemotherapy versus chemotherapy for advanced esophageal cancer: 5-year extended follow-up for the randomized phase III KEYNOTE-590 study. ESMO Open (2025) 10(12):105854. doi: 10.1016/j.esmoop.2025.105854

7. Peng Z, Zhang Y, Xu H, Yang Y, Yang M, Zhang M, et al. Camrelizumab plus CAPOX with camrelizumab based maintenance versus CAPOX alone as initial treatment for gastric or gastro-oesophageal junction adenocarcinoma: randomised phase 3 trial. Bmj (2026) 392:e086115. doi: 10.1136/bmj-2025-086115

8. Stein A, Goekkurt E, Al-Batran SE, Moosmann N, Ettrich TJ, Goetze T, et al. Perioperative pembrolizumab, trastuzumab and FLOT in HER2-positive localized esophagogastric adenocarcinoma: a phase 2 trial. Nat Med (2025) 31(12):4197-204. doi: 10.1038/s41591-025-03979-y

9. Zhang H, Huang Q, Du J, Wan X. Does adding immunotherapy to neoadjuvant chemotherapy increase postoperative morbidity in gastroesophageal junction adenocarcinoma? A propensity score-matched study. Front Immunol (2026) 17:1806803. doi: 10.3389/fimmu.2026.1806803

10. Zhou J, Guo C, Zhang M, Wang Y, Nie P, Liu X. Short-term efficacy and safety of neoadjuvant chemotherapy plus immune checkpoint inhibitors vs chemotherapy alone in locally advanced gastric cancer: a real-world propensity score-matched analysis. J Gastrointest Surg (2026) 30(7):102450. doi: 10.1016/j.gassur.2026.102450

**Figure S1**. Methodological quality of the included studies. (**A**) Risk of bias graph. (**B**) Risk of bias summary. The minus sign indicates a high risk of bias, and the plus sign indicates a low risk of bias.

**Figure S2**. Sensitivity analysis of pathological complete response rates excluding the Peng et al. trial, which compared atezolizumab plus trastuzumab plus XELOX versus trastuzumab plus XELOX in HER2-positive patients. ICIs: immune checkpoint inhibitors. RR >1 favors ICIs plus chemotherapy.

**Figure S3**. Subgroup analyses of event-free survival benefit from perioperative ICIs plus chemotherapy, stratified by PD-L1 expression: (A) CPS ≥ 1 and (B) CPS < 1. ICIs: immune checkpoint inhibitors; CPS: combined positive score. HR < 1 favors ICIs plus chemotherapy.
